# Libro de Resúmenes del XX Congreso de la Sociedad Andaluza de Neuropsicología

**DOI:** 10.31083/RN37918

**Published:** 2025-07-29

## 1. Comunicaciones Orales

**EFECTIVIDAD DE LA COMBINACIÓN DE TERAPIA LOGOPÉDICA CON ESTIMULACIÓN TRANSCRANEAL POR CORRIENTE DIRECTA EN AFASIA DE CONDUCCIÓN: UN ESTUDIO COMPARATIVO**

Noelia Boza Valencia^1^*, Cristina Gutiérrez Fernández^1^, Javier García Orza^1^

^1^Universidad de Málaga, 29071 Málaga, España

*Correspondencia: noeliabozavalencia@gmail.com

**Resumen**

La afasia de conducción se caracteriza por una producción errónea de uno o más fonemas, dando lugar a sustituciones, omisiones, inserciones y transposiciones de fonemas, que vienen acompañados de intentos por corregir los errores en la pronunciación, fenómeno conocido como *conduite d’approche* (CdA). Estas CdA pueden ser exitosas, logrando la palabra objetivo tras múltiples intentos (ej.: para “Reflujo” producir “Refujlo, reflu, reflujo”); o no exitosas, sin llegar a la palabradiana (ej.: para “Escalera” producir “Escab, escaber, escabera”). Recientemente, diferentes estudios han mostrado evidencia sobre los beneficios de la complementación de técnicas de estimulación cerebral no invasiva, como la estimulación transcraneal por corriente directa (tDCS), con la terapia logopédica tradicional, favoreciendo la reorganización neuronal en personas con afasia. El objetivo de este trabajo es presentar un caso de afasia de conducción y comparar la efectividad de la terapia logopédica combinada con dos diferentes montajes de tDCS. Las terapias se realizaron en dos momentos diferentes, con la diferencia de que en una de ellas se activó la zona de la corteza dorsolateral prefrontal (DLPFC) junto con el giro frontal inferior (IFG), mientras que en la otra sólo se estimuló ésta última zona. En ambas intervenciones se evaluó la repetición de palabras y denominación con la Western Aphasia Battery (WAB) y la tarea de repetición de pseudopalabras de Aguado. Los resultados obtenidos muestran diferencias significativas, mejorando el rendimiento del paciente, manteniéndose estos efectos durante al menos 10 días después del tratamiento. El estudio sugiere que la terapia logopédica combinada con dos montajes diferentes de tDCS tuvo un efecto positivo en la capacidad del paciente para la denominación, la repetición de palabras y de pseudopalabras, con algunas diferencias entre montajes que sugieren que la activación añadida de DLPFC, un área relevante para el control cognitivo, podría suponer beneficios adicionales.

**Palabras Clave**

afasia de conducción; conductas de aproximación; estimulación transcraneal por corriente directa; terapia logopédica

**TEORÍA DE LA MENTE EN NIÑOS SORDOS Y OYENTES DE 6 A 12 AÑOS**

Laura Fernández García^1,2^*, Nahuel Gioiosa Maurno^1,2^, Jessica Phillips-Silver^3^, María Teresa Daza González^1,2^

^1^Centro de Investigación para el Bienestar y la Inclusión Social, 04120 Almería, España

^2^Departamento de Psicología, Almería, España

^3^Growing Brains, Washington, D.C., USA

*Correspondencia: lfg403@ual.es

**Resumen**

**Introducción y Objetivos**: Una de las Funciones Ejecutivas que parece verse afectada a causa de la privación auditiva es la Teoría de la Mente (ToM). El uso de dispositivos de audición, como el implante coclear facilitan el acceso al sonido en la población con sordera. Por lo que en este estudio se plantea un doble objetivo: (1) Estudiar la capacidad de comprensión de falsas creencias (una de las habilidades claves de la ToM) en un grupo de niños sordos en comparación con sus pares oyentes. Y, (2) explorar el efecto del uso de implante coclear en dicha habilidad de ToM en los niños sordos. **Método**: Participaron un total de 41 niños sordos y oyentes de entre 6 y 11 años (Edad media = 9,07, DT = 1,68, 46,34% niñas) quienes realizaron una versión adaptada de la tarea de Shally y Anne incluida en la “*Executive Brain Battery*” (EBB). **Resultados y Conclusiones**: Los resultados mostraron diferencias estadísticamente significativas entre ambos grupos de audición, donde los niños sordos tuvieron más dificultades para comprender falsas creencias. Además, también se observó un efecto principal del nivel educativo de los padres, lo que refleja que un nivel educativo más bajo estaba asociado con una ejecución más pobre en dicha tarea. Respecto al uso de implante coclear, parece mostrar un efecto negativo en la comprensión de falsas creencias en el grupo de niños sordos incluidos en este estudio. Estos resultados son congruentes con los observados previamente en la literatura, donde se ha observado que las dificultades en esta habilidad de ToM en los niños sordos podría estar relacionada con una menor exposición a diálogos referentes a los estados emocionales y mentales dentro del contexto familiar.

**Palabras Clave**

teoría de la Mente; sordera; funciones ejecutivas; nivel educativo de los padres

**PROGRAMA DE ESTIMULACIÓN NEUROPSICOLÓGICA PARA MENORES VULNERABLES DE LOS SUBURBIOS DE CIUDAD DE GUATEMALA**

Pablo Rodríguez-Prieto^1,2^, Ian Simpson^3^, 41704 Joaquín Ibáñez-Alfonso^1,2^*

^1^Laboratorio de Neurociencia Humana, Departamento de Psicología, Universidad Loyola Andalucía, Dos Hermanas, 14004 Sevilla, España

^2^Instituto de Desarrollo de la Universidad Loyola Andalucía, Fundación ETEA, 18071 Córdoba, España

^3^Departamento de Psicología Experimental, Universidad de Granada, Granada, España

*Correspondencia: jaibanez@uloyola.es

**Resumen**

**Introducción**: Guatemala está entre los países más pobres de américa latina, con elevados niveles de desigualdad social, pobreza y violencia extrema. Este contexto socioeconómico desfavorable supone un riesgo para el desarrollo cognitivo y emocional en menores. Además, el empeoramiento de la estabilidad del país debido a la pandemia de COVID-19 ha agravado la brecha socioeconómica que afrontan sus habitantes. Este trabajo presenta los resultados de la implementación de un programa de estimulación cognitiva y emocional con el que se pretende mejorar el rendimiento académico y calidad de vida de estos menores. **Metodología**: Se realizó un ensayo controlado aleatorizado para evaluar la efectividad de un programa de estimulación cognitiva y emocional de 24 sesiones con actividades de Atención, Lenguaje, Funciones Ejecutivas, y Cognición Social, a través de la plataforma de neurorrehabilitación NeuronUP. Se dividió a participantes de 5 º de Primaria (n = 464) aleatoriamente en dos grupos: (1) Experimental, que recibió la intervención, y (2) Control, que realizó actividades de refuerzo de lectura. Se realizaron evaluaciones pre- y post-intervención, y seguimiento posterior al año con intercambio de roles entre grupo experimental y control. **Resultados**: El grupo que estaba recibiendo la intervención cognitiva mostró un mayor promedio de mejoría, viéndose resultados más elevados en participantes con trastornos del neurodesarrollo. Sin embargo, el resultado mayor relevancia fue que la nota media del grupo que recibía la intervención se mantenía durante el año, mientras que la del grupo que realizaba sólo el programa de lectura decrecía de forma significativa. **Discusión**: Los resultados parecen indicar que el programa de estimulación actuó como un factor protector significativo para el rendimiento académico de los participantes tras haber pasado dos años sin escolarización regulada durante la pandemia, implicando una posible mejoría en su calidad de vida.

**Palabras Clave**

neuropsicología; desarrollo infantil; vulnerabilidad; estimulación cognitiva; competencia emocional; ensayo aleatorio controlado

## 2. Pósteres

**DÉFICIT NEUROCOGNITIVO MULTIDOMINIO POR AFECTACIÓN FRONTO- TEMPORAL IZQUIERDA. PRESENTACIÓN DE UN CASO**

Silvia Hidalgo Berutich^1,2^*, Bibiana Villalta Álvarez^1,2^*

^1^Centro de psicología AVANZA, Universidad de Málaga, 29001 Málaga, España

^2^Centros de Atención Infantil Temprana de la Fundación la Esperanza E.B.S., 29140 Málaga, España

*Correspondencia: shidalgo@avanza-online.es; bibianavillalta@hotmail.com

**Resumen**

La esclerosis temporal mesial (ETM) es la pérdida neuronal y gliosis en el hipocampo, con cierta reorganización de las vías neuronales y formación de un foco epileptógeno. Las regiones mesiales temporales son cruciales para las funciones ejecutivas, especialmente la memoria. También hay afectación del sistema límbico. Se presenta un estudio neuropsicológico basado en la evidencia. Descripción del caso: adulta de 50 años con pérdida de capacidad cognitiva crónica que afecta a su funcionalidad en AVD. Los primeros síntomas surgieron a los 19 años produciendo una pérdida significativa de capacidad cognitiva. Recibió tratamiento psicofarmacológico y neurorrehabilitación recuperando gran parte de la capacidad hasta la actualidad. Sintomatología principal: prosopagnosia, pérdida de memoria, incapacidad de sentir temperatura corporal, pérdida de lívido, inapetencia, adipsia, pérdida de capacidad lectora, problemas de visión sin afectación visual, desorientación temporal, dificultad en praxias motoras, labilidad emocional. Se ha realizado una valoración ecológica contextualizada a la sintomatología presente. Pruebas: Entrevista Clínica Semiestructurada, MINI-MENTAL examen cognoscitivo, Cuestionario cognitivo (SPMSQ), Test de símbolos y Dígitos (SDMT), Test Visoconstructivo (BENDER), Test de 5 dígitos (FDT), Test MATRICES, Índice de Actividades Instrumentales de Vida Diaria y Escala de Depresión y Ansiedad. Resultados: se detectan alteraciones en las Funciones Ejecutivas frías (atención, memoria verbal, memoria de trabajo, procesamiento, planificación y flexibilidad), trastorno del lenguaje, trastorno de percepción visual. Junto con alteraciones en Funciones Ejecutivas Cálidas (regulación emocional, toma de decisiones). Funciones Ejecutivas preservadas: capacidad intelectual, reserva cognitiva, atención focalizada, razonamiento lógico, conciencia del problema y personalidad. Intervención: programa de neurorehabilitación basado en el perfil neurocognitivo de la paciente con puntos débiles (o alteraciones de las frías y Cálidas detectadas) y puntos fuertes (o Funciones Ejecutivas preservadas). Conclusión: la paciente muestra más dificultad en recuperar FFEE frías que en la FFEE cálidas, necesitando aumentar el número de sesiones de intervención para poder recuperar las AVD.

**Palabras Clave**

déficit neurocognitivo; mesial; temporal; neuropsicología; funciones ejecutivas

**NEURODIVERGENCIA EN JÓVENES ADULTOS 18–30 AÑOS CON DIVERSIDAD FUNCIONAL. EL MÉTODO AVANZA**

Silvia Hidalgo Berutich^1^

^1^Centro de Psicología AVANZA, Universidad de Málaga, 29001 Málaga, España

*Correspondencia: shidalgo@avanza-online.es

**Resumen**

**Introducción**: Presentamos un trabajo centrado en la NEURODIVERGENCIA en jóvenes adultos 18–30 años con diversidad funcional mediante la aplicación del método AVANZA. Esta metodología promueve una aplicación integradora de la NEUROPSICOLOGIA, interviniendo en las competencias personales y socio-emocionales. **Objetivo**: Entrenamiento en la AUTOEFICACIA PERCIBIDA para lograr alcanzar los hitos evolutivos de este periodo de transición de la adolescencia a la adultez. El papel del clínico es guía-asesor, otorgando al joven una actitud proactiva y de protagonismo en la terapia. **Metodología**: Aplicación del enfoque transdisciplinar. Procedimientos: psicología clínica tradicional, terapias de tercera generación (TTG) y estrategias neuropsicológicas basada en neuroafectividad. Participantes: 10 jóvenes adultos de 19 a 30 años; 4 mujeres y 6 varones. Instrumentos de evaluación: Entrevista motivacional; Historia de vida; FDT; MATRICES; D2-R; Cuestionario Personalidad TAMADUL; Cuestionario Ansiedad-Depresión CECAD y Criterios DSM5. En la evaluación se detectaron perfiles clínicos de TDAH, TOC, DISLEXIA. Intervención. Aplicación del Método AVANZA que consiste en: diseño de perfiles neurocognitivos mapeando las FFEE fuertes/débiles; entrenamiento con Técnicas de TTG (como Terapia Aceptación y Compromiso, ACT y Terapia Metacognitiva, TMC) y técnicas neuroafectivas (como Mentalización de Emociones). **Resultados**: Multifuente: análisis DAFO, encontrando Dificultades (limitaciones emancipación fuera del hogar familiar); Amenazas (escasez de ofertas laborales); Fortalezas (implicación en la terapia); y Oportunidades (la regulación emocional). De los autoregistros administrados durante la terapia, se obtiene: reducción de síntomas de ansiedad-depresión; de respuestas de evitación social y de pensamientos automáticos negativos. Persisten dificultades en: autoestima, planificación e inhibición conductual. **Conclusión**: La neuropsicología ofrece una perspectiva integradora efectiva para atender las peculiaridades del desarrollo del cerebro del/a joven adulto neurodivergente, interviniendo en las Funciones Ejecutivas alteradas/preservadas. Contribuye al autoconocimiento y bienestar psicosocial de esta población emergente y posibilita mecanismos psicológicos para afrontar las AVD como paso previo al desarrollo del PROYECTO DE VIDA en la adultez.

**Palabras Clave**

Jóvenes-adultos; neurodivergencia; autoeficacia percibida; Método AVANZA; Transdisciplinariedad

**EFECTO DEL APRENDIZAJE COOPERATIVO EN EL DESARROLLO DEL PENSAMIENTO CREATIVO Y LA RIQUEZA LÉXICA: ESTUDIO LONGITUDINAL EN EL CONTEXTO ESCOLAR**

Rafael Segundo-Marcos^1,2^*, María Teresa Daza González^1,2^, Verónica López Fernández^3^

^1^Departamento de Psicología, Universidad de Almería, 04120 Almería, España

^2^Centro de Investigación para el Bienestar y la Inclusión Social (CIBIS), Universidad de Almería, 04120 Almería, España

^3^Departamento de Psicología y Psicobiología de la Educación, Universidad Internacional de La Rioja (UNIR), 26006 Logroño, España

*Correspondencia: rsm556@ual.es

**Resumen**

El aprendizaje cooperativo es una metodología educativa que organiza a los estudiantes en grupos heterogéneos para alcanzar objetivos comunes, mejorando las interacciones sociales y potenciando el desarrollo de ciertos procesos y habilidades cognitivas. Este estudio longitudinal investiga el impacto del aprendizaje cooperativo en el desarrollo del pensamiento creativo y la riqueza léxica en niños de 9 a 12 años, comparándolo con un método de aprendizaje individualista. Adicionalmente, este estudio analiza la relación entre ambas variables. Participaron cincuenta y siete estudiantes de dos escuelas de Educación Primaria, divididos en dos grupos: uno bajo la metodología cooperativa y otro bajo metodología individualista. Se evaluó el pensamiento creativo y dos dimensiones de riqueza léxica (diversidad y densidad léxica) en tres momentos distintos durante dos cursos escolares. Los resultados mostraron diferencias en el desarrollo del pensamiento creativo entre ambos grupos El grupo cooperativo experimentó mejoras consistentes entre las tres evaluaciones mientras que el grupo individualista solo mostró cambios significativos entre la segund y la tercera evaluación. En cuanto a la densidad léxica, se observaron tendencias diferenciales en su desarrollo, siendo significativas en el grupo cooperativo a lo largo de las tres evaluaciones. Por último, el análisis de la diversidad léxica reveló algunas diferencias entre los grupos, aunque el progreso fue generalmente más estable y similar entre ambos grupos. Finalmente, se observaron correlaciones entre el pensamiento creativo y las dos dimensiones de riqueza léxica: densidad y diversidad léxica. Estos hallazgos resaltan la influencia del entorno educativo en el desarrollo del pensamiento creativo y las habilidades lingüísticas, destacando la importancia de las metodologías de enseñanza como factores clave en el desarrollo de estos procesos y subrayando las implicaciones de la riqueza léxica en el potencial de pensamiento creativo.

**Palabras Clave**

pensamiento creativo; riqueza léxica; aprendizaje cooperativo; contexto escolar

**COGNICIÓN SOCIAL Y PSICOPATOLOGÍA EN PERSONAS CON TRASTORNOS POR USO DE SUSTANCIAS EN TRATAMIENTO AMBULATORIO**

Elena Serrano Jiménez^1,2^*, Esperanza Vergara Moragues^3^, Adolfo Piñón Blanco^1,4^, Carlos Spuch^1^

^1^Translational Neuroscience Research Group, Galicia Sur Health Research Institute (IIS-Galicia Sur), SERGAS-UVIGO, CIBERSAM, 36312 Vigo, España

^2^Universidad Complutense de Madrid, 28040 Madrid, España

^3^Universidad de Cádiz, 11001 Cádiz, España

^4^Unidad Asistencial de Drogodependencias CEDRO, 36211 Vigo, España

*Correspondencia: eleser01@ucm.es

**Resumen**

**Introducción**: La cognición social (CS) es entendida como un proceso cognitivo que permite elaborar conductas sociales adaptativas, siendo sus dominios más estudiados el reconocimiento de emociones y la teoría de la mente. Una adecuada CS parece estar asociada a una mejor respuesta terapéutica en personas con trastornos por uso de sustancias (TUS). La presencia de patología dual, coexistencia en una persona de un TUS con otras patologías psiquiátricas, podría estar relacionada con mayores alteraciones en la CS, lo que a su vez conlleva menor adherencia al tratamiento y mayor número de recaídas . El objetivo de este estudio es evaluar la CS y la patología dual en personas con TUS. **Método**: Diseño transversal descriptivo. La muestra estuvo compuesta por 11 personas con TUS que acuden a tratamiento ambulatorio. Se evaluaron los dominios de CS de procesamiento emocional, con el Ekman 60 Faces Test (FEEST), y la empatía, con el Interpersonal Reactivity Index (IRI). Para evaluar la patología dual se utilizaron la Entrevista de Cribado de Patología Dual (ECDD) y el Cuestionario International Personality Disorder Examination (IPDE). **Resultados**: Se encontraron alteraciones en el reconocimiento de emociones (27,2%), especialmente en asco (54,5%) y miedo (36,4%). También se encontró alterada la subescala de angustia personal del IRI (36,4%). La ECDD y el IPDE mostraron una alta probabilidad de presencia de depresión (90,9%), ansiedad generalizada (81,8%), manía (81,8%), trastorno de personalidad evitativo (81,8%) y trastornos de estrés postraumático (63,6%). **Conclusiones**: Los datos indican una presencia de alteraciones en el procesamiento emocional y en la empatía en personas con TUS. Asimismo, el cribado de patología dual muestra unos porcentajes elevados en la muestra evaluada. Por ello, sería necesario tener presente la evaluación de la CS y psicopatología de estas personas y estudiar su relación con el éxito y diseño del tratamiento.

**Palabras Clave**

adicción; trastorno por uso de sustancias; patología dual; cognición social; empatía; reconocimiento de emociones

**INTRAOPERATIVE PROTOCOLS FOR ASSESSING COGNITIVE, EMOTIONAL, AND SOCIAL FUNCTIONES DURING AWAKE BRAIN SURGERY IN PATIENTS WITH RIGHT HEMISPHERE LESIONS: A SYSTEMATIC REVIEW**

Laura Amores-Carrera^1^*, David Sabsevitz^2^, Guillaume Herbet^3,4,5,6^, Isabel Martín-Monzón^1^

^1^Department of Experimental Psychology, Faculty of Psychology, University of Seville, 41004 Seville, Spain

^2^Department of Psychiatry and Psychology, Division of Neuropsychology, Mayo Clinic Florida, Jacksonville, FL 32224, USA

^3^Department of Neurosurgery, Gui de Chauliac Hospital, 34295 Montpellier, France

^4^Praxiling Lab, UMR5267 CNRS & Paul Valéry University, Bâtiment de Recherche Marc Bloch, 34090 Montpellier, France

^5^Department of Medicine, University of Montpellier, Campus ADV, 34095 Montpellier, France

^6^Institut Universitaire de France, 75231 Paris, France

*Correspondencia: lacarrera@us.es

**Resumen**

**Introduction**: The right hemisphere plays a critical role in cognitive, emotional, and social functions. Awake craniotomy task selection and establishing the patient’s preoperative cognitive baseline on these tasks are essential for ensuring the reliable mapping of Function and for avoiding postoperative functional deficits. While neuropsychological deficits post-right hemisphere resections are well-documented, literature on neuropsychological protocols suitable during awake brain surgery mapping is significantly lacking. **Objective**: To address this gap in the literature, we conducted a systematic review focusing on cognitive and emotional processes associated with the right hemisphere. Our review also analyzed the primary neuropsychological tasks used for mapping the right hemisphere during awake brain surgery in patients with brain tumors. **Methods**: The scientific databases used were PubMed, PsycINFO, Scopus, and the Cochrane Library. A total of 365 relevant studies were identified from 2005 to March 2024. Following the selection process, 31 articles that met the inclusion and exclusion criterio were comprehensively reviewed. **Resultados**: The main finding concerns the cognitive processes assessed and intraoperative tasks commonly used to evaluate visuospatial processing, language and speech, executive functions, social cognition, and motor/sensory functions in the right hemisphere. Thus, the most used tests to assess language and speech functions in patients with right hemisphere lesions included the naming task and the PPTT. Spatial cognition was mainly evaluated with the line bisection task, while social cognition was assessed using the RME test. Motor and sensory functions were frequently evaluated through dual-tasking and upper and lower limb movement assessments and executive functions were typically assessed with the N-back test and Stroop test. **Conclusions**: This is the first comprehensive review aimed at guiding multidisciplinary teams in right hemisphere brain tumor surgery, focusing on identifying vulnerable cognitive functions and standard awake brain mapping methods.

**Palabras Clave**

awake brain surgery; intraoperative mapping; right hemisphere; neuropsychological protocol; connectome

**GESTANTES SIN PAREJA POR DECISIÓN PROPIA VERSUS GESTANTES CON PAREJA: DOFERENCIAS EN ESTRÉS Y PSICOPATOLOGÍA**

Natalia Bueso-Izquierdo^1^*, Marina Martín Cabello^1^, M. Isabel Peralta-Ramírez^1^

^1^Facultad de Psicología, Departamento: Personalidad, Evaluación y Tratamiento, Universidad de Granada, 18071 Granada, España

*Correspondencia: nbueso@ugr.es

**Resumen**

**Introducción**: Hay un modelo de familia que está creciendo en nuestra sociedad y es el de madres sin pareja por decisión propia (MSPDP). Son mujeres que libremente deciden gestar y/o criar a su hijo/a o hijos/as sin la corresponsabilidad de una pareja. Aunque es una configuración familiar en constante crecimiento, son pocos los trabajos relacionados con la salud mental de estas mujeres y su efecto en el desarrollo de sus menores. A pesar de la alta prevalencia del estrés durante el embarazo, los trastornos mentales en el embarazo son de las condiciones menos evaluadas. El estrés perinatal, conlleva repercusiones, donde los cambios ocurridos en el eje Hipotalámico-Pituitario-Adrenal durante el embarazo producen alteraciones en la futura madre y el feto. **Objetivo**: Comparar un grupo de mujeres gestantes sin pareja por decisión propia con un grupo de mujeres gestantes con pareja, para comprobar si existen diferencias en los niveles de estrés y síntomas psicopatológicos entre ellas. **Método**: 91 mujeres: 45 gestantes con pareja (GCP) y 46 gestantes sin pareja por decisión propia (GSPDP). **Resultados**: No existen diferencias entre ambos grupos en los niveles de estrés específico del embarazo ni en los niveles de estrés percibido. Por otro lado, las mujeres embarazadas sin pareja por decisión propia presentan menos síntomas psicopatológicos que aquellas mujeres embarazadas que tienen pareja. **Conclusiones**: Ser gestante sin pareja por decisión propia, no conlleva tener más probabilidades de desarrollar problemas psicológicos, no solo en términos de estrés percibido, ansiedad y depresión, sino también en estrés específico del embarazo. Gracias a esta investigación se aumenta la relevancia sobre este modelo de familia, visibilizando otras maneras de gestar, que hasta hace algunos años estaba “mal visto” por parte de la sociedad y poco valorado en general.

**Palabras Clave**

gestantes sin pareja; estrés; embarazo; psicopatología

**DESARROLLO DEL LÓBULO FRONTAL EN ESCOLARES CON ANTECEDENTES DE PREMATURIDAD Y MUY BAJO PESO AL NACER. ESTUDIO DE RESONANCIA MAGNÉTICA**.

Marta Elena Carrasco Solís^1,2^*, Ana Nieto Ruiz^3,4^, Ana Campos Martínez^5^, Aida Ruiz^5^, Ana Cadenas Villegas^5^, Emilia Vélez Moreno^5^, Cristina Alcaide Baena^5^, Pilar Ureña^5^, Ainhoa Rodrigo^5^, Paloma Garcia Peces^5^, Carolina Laynez Rubio^1,2^, José Uberos Fernández^2,5^

^1^Unidad de Neuropediatría, Servicio Pediatría, Hospital Universitario Clínico San Cecilio, 18007 Granada, España

^2^Facultad de Medicina, Universidad de Granada, 18071 Granada, España

^3^Departamento de Metodología de las Ciencias del Comportamiento, Facultad de Psicología, Universidad de Granada, 18071 Granada, España

^4^EURISTIKOS, Centro de Excelencia para la Investigación Pediátrica, Centro de Investigación Biomédica, 18016 Granada, España

^5^Unidad de cuidados intensivos Neonatales, Hospital Universitario Clínico San Cecilio, 18007 Granada, España

*Correspondencia: martaecs.psicologia@gmail.com

**Resumen**

**Introducción**: El crecimiento fetal restringido (RGF) es resultado de un aporte insuficiente de oxígeno y nutrientes en el feto durante un periodo gestacional de máxima vulnerabilidad neurológica y donde pueden producirse alteraciones y déficits en el desarrollo neurológico. Los recién nacidos muy prematuros con RGF muestran, según algunos estudios, una maduración neuronal acelerada en las regiones caudales del tronco encefálico, pero una maduración neuronal moderadamente retrasada en las regiones rostrales. **Objetivos**: Analizar el desarrollo de las áreas corticales y subcorticales frontales en niños con antecedente de prematuridad y muy bajo peso al nacer y evaluar su relación con el antecedente de RGF y el peso del recién nacido. **Método**: Estudio de cohortes, realizado en dos etapas: retrospectiva, realizado en recién nacidos de muy bajo peso nacidos en nuestro hospital entre enero de 2008 y diciembre de 2016; y prospectiva donde se realiza un análisis estructural cerebral mediante resonancia magnética cerebral. **Resultados**: Se realizó estudio de resonancia magnética cerebral en 48 niños de 9,2 años (95% CI 8,5–9,9) con antecedente de prematuridad y peso al nacer menor de 1500 g. De ellos, 9 presentaron además RGF. La RGF se asocia con áreas de corteza orbitofrontal medial, pars opercularis y pars triangularis significativamente más pequeñas, en tanto la corteza anterior del cíngulo fue significativamente mayor. **Conclusiones**: La RGF y el peso al nacer son factores relacionados con alteraciones estructurales en el lóbulo frontal. El desarrollo del cortex orbitofrontal y la para triangularis se ven fuertemente influenciadas por el peso del recién nacido al nacer.

**Palabras Clave**

lóbulo frontal; prematuridad; bajo peso al nacer; crecimiento fetal restringido; imágenes de resonancia magnética

**ALTERACIONES CEREBRALES Y PSICOPATOLÓGICAS EN MUJERES SUPERVIVIENTES DE VIOLENCIA DE GÉNERO**

Cristina Jiménez Terrón^1^*, Julia Daugherty^1^, María Pérez^1^, Natalia Hidalgo^1^, Juan Verdejo Román^1^, Miguel Pérez García^1^

^1^Centro de Investigación Mente, Cerebro y Comportamiento, Universidad de Granada, 18071 Granada, España

*Correspondencia: cristinajmtr@hotmail.es

**Resumen**

Más de una cuarta parte de las mujeres de entre 15 y 49 años que ha estado en una relación informa haber sufrido algún tipo de violencia por parte de su pareja. Actualmente se sabe que la exposición continua al maltrato tiene consecuencias tanto físicas, como psicológicas y aunque existe información sobre las consecuencias psicológicas, la cerebrales aún no están claras. El objetivo de este estudio fue conocer las diferencias psicopatológicas y cerebrales entre un grupo de mujeres supervivientes de violencia de género y un grupo de controles que no habían sufrido esta violencia. Los resultados obtenidos muestran diferencias significativas entres los grupos en ansiedad generalizada, depresión mayor, severidad de la violencia de género, calidad de vida y eventos traumáticos en la infancia. Por otro lado, también encontramos diferencias específicas en la sustancia gris, siendo menor para las mujeres supervivientes en el lóbulo temporal izquierdo. Además, se observó una correlación positiva entre la cantidad de materia gris en esta región y las puntuaciones en calidad de vida y ansiedad generalizada. Estos resultados al ser preliminares deberán ser replicados en futuros estudios para conocer cuál es la relación entre la reducción en materia gris del lóbulo temporal izquierdo y la psicopatología de las mujeres.

**Palabras Clave**

violencia de género; mujeres supervivientes; alteraciones cerebrales y psicopatología

**EFECTO DE LA TÉCNICA DE REPETICIÓN ESPACIADA EN LA CAPACIDAD DE APRENDIZAJE EN PACIENTES CON DEMENCIA: REVISIÓN SISTEMÁTICA CON META-ANÁLISIS**

María Dolores de la Rosa Gámiz^1,2^*, Jesús González Moreno^1^, María García Cantero^1^

^1^Universidad Internacional de Valencia, 40062 Valencia, España

^2^Universidad Europea de Madrid, 28670 Madrid, España

*Correspondencia: delarosagamiz@gmail.com

**Resumen**

La técnica de repetición espaciada ha mostrado un efecto beneficioso en la capacidad de aprendizaje en pacientes con déficit de memoria de leve a moderado. Sin embargo, los datos existentes muestran importantes incongruencias con respecto a la generalización y mantenimiento a largo plazo de dicho efecto. Por lo tanto, este trabajo presenta una revisión sistemática con meta-análisis sobre la evidencia existente del efecto de la repetición espaciada en la retención y recuperación de la información aprendida en pacientes con demencia en comparación a otras técnicas de aprendizaje. Además, queríamos analizar si el efecto se mantiene a largo plazo y generaliza a otras variables o contextos. Entre noviembre de 2020 y enero de 2021, se realizó una búsqueda sistemática de ensayos controlados aleatorizados en PUBMED, MEDLINE, Web of Science, PubPsych, y ProQuest. Los resultados de 10 estudios mostraron que el efecto positivo de la repetición espaciada sobre el aprendizaje de personas con deterioro de la memoria no difería significativamente del efecto de otras técnicas de aprendizaje (p. ej., método de aprendizaje sin error); el efecto incluso aumenta significativamente en combinación con otras técnicas en comparación a la aplicación aislada. Sin embargo, los escasos datos sobre el efecto a largo plazo fallan en mostrar resultados positivos, mientras que la generalización a otras variables o contextos depende de la similitud con el material/contexto entrenado. Por lo tanto, esta revisión sistemática muestra que la evidencia sobre el efecto de la repetición espaciada en pacientes con déficits de memoria es aún escasa y se basa en estudios de calidad metodológica moderada. En el futuro, sería importante priorizar una mayor validez científica en la investigación de las técnicas de aprendizaje en pacientes con demencia con la finalidad de conocer la mejor opción de aplicación en el ámbito clínico.

**Palabras Clave**

repetición espaciada; enfermedad de Alzheimer; demencia; aprendizaje; memoria

**MÉTODO DENVER COMO RECURSO NEUROPSICOLÓGICO PARA MEJORAR FUNCIONES EJECUTIVAS EN TEA. PRESENTACIÓN DE CASO CLÍNICO**

Silvia Hidalgo Berutich^1^*, Clara Gaona Galacho^1^, María Canca Román^1^

^1^Centro de Psicología AVANZA, Universidad de Málaga, 29071 Málaga, España

*Correspondencia: shidalgo@avanza-online.es

**Resumen**

El Método Denver tiene como objetivo reducir la gravedad de las manifestaciones del trastorno del espectro autista (TEA) y promover una adecuada evolución en las actividades de la vida diaria del menor, interviniendo en tres áreas esenciales del neurodesarrollo infantil: comunicativa, cognición y socioemocional. En este trabajo se presenta un caso basado en la evidencia (N = 1): niña de 7 años con diagnóstico pluripatológico de discapacidad intelectual profunda, síndrome disfórmico y crisis de epilepsia nocturna que cursa con síntomas trastorno del espectro autista. Evaluación: Inventario de Desarrollo BATTELLE, Currículum Denver y Rueda del Desarrollo proporcionando información sobre el perfil neurocognitivo de la menor. Intervención: programa de Neurorrehabilitación AVANZA centrado en la neuroestimulación de Funciones Ejecutivas (FFEE) preservadas y deficitarias que presenta la menor en relación a las actividades de la vida diaria (AVD), mediante 2 sesiones individuales semanales y 1 sesión de psicoeducación con los padres para generalizar el aprendizaje de AVD. Aplicación del Método AVANZA, que integra los hitos evolutivos alcanzados por la menor y los que están pendientes de lograr tomando como referencia el Método Denver junto con tareas multisensoriales “ad hoc” para activar en la menor los canales neurosensitivos como vías principales de aprendizaje. FFEE a potenciar: control conductual, atención sostenida, praxias, procesamiento de la información, cognición y memoria de trabajo. Resultados: mejoría en el área comunicativa, cognitiva y socioemocional tras la aplicación de la intervención durante 36 meses. Aspectos a mejorar: generalización de aprendizajes a otros contextos de desarrollo de la menor.

**Palabras Clave**

método denver; neurorrehabilitación; perfil neurocognitivo; FFEE; AVD

**PERFIL COGNITIVO DE PACIENTES QUE EMERGEN DEL ESTADO DE MÍNIMA CONCIENCIA: UNA REVISIÓN SISTEMÁTICA**

Santiago Marín-Balcázar^1^*, Samuel López-Rodríguez^1^, Joaquín Ibáñez-Alfonso^1^*, Enrique Noé-Sebastián^2^, Alejandro Galvao-Carmona^1,2^*

^1^Departamento de Psicología y Laboratorio de Neurociencia Humana, Facultad de Psicología y Educación, Universidad Loyola Andalucía, 41704 Sevilla, España

^2^IRENEA-Instituto de Rehabilitación Neurológica, Fundación Vithas, 46007 Valencia, España

*Correspondencia: ssantiagomarin@al.uloyola.es; jaibanez@uloyola.es; agalvao@uloyola.es

**Resumen**

**Introducción**: Las personas en estado de mínima consciencia (EMC) muestran una autoconciencia fluctuante y un comportamiento y una capacidad de respuesta inconsistentes respecto a su entorno, lo que complica la elucidación de los mecanismos cognitivos que subyacen a su emergencia. Actualmente, la definición de emergencia en EMC es controvertida, caracterizándose por la comunicación interactiva funcional o el uso funcional de dos objetos diferentes. **Objetivo**: Revisar sistemáticamente el perfil cognitivo de las personas que emergen del EMC (eEMC). **Método**: La revisión sistemática se desarrolló según las normas PRISMA. Los artículos seleccionados se obtuvieron en una búsqueda bibliográfica en dos bases de datos mediante una cadena de búsqueda (minimally conscious state OR disorder of consciousness) AND (emergence) AND (Neuropsy* OR cognit* OR attention OR memory OR executive functions OR perception OR language OR assessment). Los artículos cumplieron los criterios de inclusión y exclusión para su inserción en la revisión. La búsqueda primaria generó 1892 artículos: 1713 de PubMed y 179 de WOS. A continuación, se excluyeron 132 duplicados, analizando 1760 artículos. Luego, se excluyeron 1604 artículos mediante la comprobación del título y el resumen. Posteriormente, se revisaron a texto completo los 71 restantes. **Resultados**: Sólo cuatro artículos cumplían los criterios. Los pacientes con eMCS presentaron puntuaciones significativas en el CRS-R en relación con el uso de objetos funcionales y/o la comunicación funcional. Además, esta población presentaba una discapacidad moderada a extremadamente grave evaluada mediante la escala DRS, lo que implicaría que tras la emergencia presentaban una dependencia total según el índice de Barthel y la medida de dependencia funcional. **Conclusiones**: Los pacientes que salen de un eEMC presentan comunicación funcional (capacidad verbal/lenguaje) y/o búsqueda funcional de objetos (capacidad motora/praxias) y procesos atencionales. No obstante, algunos pacientes manifestaron una discapacidad severa con deterioro funcional. Por último, no hay muchos estudios que investiguen otras funciones cognitivas, con sólo cuatro artículos elegibles para su inclusión en esta revisión.

**Palabras Clave**

estados alterados de conciencia; procesos cognitivos; emergencia; estado de mínima consciencia

**PERFIL NEUROPSICOLÓGICO EN UN CASO DE SÍNDROME DE CAPGRAS**

Aarón Fernández-Del Olmo^1,2,3^*, Marta Usua-Calabuig^4^

^1^ISANEP, Alcalá de Guadaira, 41500 Seville, España

^2^Universidad Internacional de la Rioja (UNIR), 26006 Logroño, España

^3^Universidad Loyola Andalucia, 41014 Sevilla, España

^4^Neuro-Crecer, Sevilla, España

*Correspondencia: aaronneuropsicologia@gmail.com

**Resumen**

El síndrome de Capgras es un trastorno neuropsiquiátrico que en un 25–50% de las ocasiones aparece en el contexto de algún tipo de demencia. Se presenta el caso de una mujer de 79 años, diesta, y sin antecedentes neurológicos previos, que comienza a mostrar 8 meses antes la idea de que su marido ha sido secuestrado y cambiado por una persona exactamente igual que ahora vive con ella. A ese delirio le acompaña la idea de que su casa ha sido ampliada con otras partes que no se corresponden, como formando un hotel. 4 meses después este delirio se extiende también a una de sus hijas. Una exploración neurológica previa señala la presencia de una lesión hipodensa en hemisferio cerebeloso derecho sin expresión clínica reseñable. Se procede a evaluación neuropsicológica para hacer un diagnóstico diferencial de un posible cuadro degenerativo subyacente. Se elabora protocolo con pruebas para atención, memoria, funciones ejecutivas, percepción visual y función práxica. El resultado de las pruebas muestra un perfil con una clara alteración de la cognición espacial y leves alteraciones a nivel atencional y ejecutivo, mostrándose preservado el lenguaje salvo por leves problemas en la articulación. Se discute la posibilidad las ideas delirantes se encuentren en un contexto de enfermedad neurodegenerativa no detectada previamente y su posible compatibilidad con una demencia por cuerpos de Lewy o una forma de parkinsonismo.

**Palabras Clave**

delirio de capgras; cognición espacial; cuerpos de Lewy; deterioro cognitivo

**EXPLOSIÓN LINGÜÍSTICA INESPERADA: UN CASO DE ADQUISICIÓN LATENTE DEL LENGUAJE EN UN NIÑO PREMATURO**

María del Pilar Garrido Borrego^1,2^*, Miguel Cruz^1,2^, Aaron Fernández del Olmo^1,2,3^

^1^Centro ISANEP, Alcalá de Guadaira, 41500 Sevilla, España

^2^Universidad Internacional de la Rioja, 26006 Logroño, España

^3^Universidad Loyola Andalucía, 41014 Sevilla, España

*Correspondencia: pgarridoborrego.neuropsicolo@gmail.com

**Resumen**

Presentación de un caso clínico. Paciente que actualmente cursa con 6 años y 7 meses (junio 2024), acude a terapia logopédica desde 2022 y terapia neuropsicológica al inicio del presente año. Nacimiento pretérmino (30+1 semanas) en contexto de embarazo gemelar, leucomalacia periventricular, APGAR 3/5, posible riesgo neurológico por prematuridad y bajo peso. Durante sus primeros seis años de vida no utiliza expresiones lingüísticas pese al tratamiento realizado en CAIT y es trabajado desde el área de logopedia con sistemas de comunicación alternativa. Se realiza evaluación neuropsicológica a la edad de 6 años y 7 meses a petición de la familia ante la aparición súbita de palabras de poca longitud que designan a personas y familiares unos meses antes. Se utiliza un protocolo compuesto por subpruebas Nepsy-II, subpruebas de la batería Eni, y el test K-bit. Se objetiva una. Puntuación global en K-BIT de 94 (medio), destacando por encima de lo esperado para su edad la escala de vocabulario Pt: 117 (Alto), siempre atendiendo a los problemas articulatorios que presenta durante las producciones verbales. Este hecho puede ser un indicador de que, pese a la prematuridad y el perfil acontecido en el desarrollo, el lenguaje se ha adquirido de forma latente, lo que ha permitido que haya surgido una explosión del lenguaje, por lo que sería recomendable valorar el lenguaje más allá de las verbalizaciones intencionales en niños prematuros para poder calibrar su desarrollo real.

**Palabras Clave**

Prematuridad; trastorno socio comunicativo; lenguaje; neuropsicología infantil; estimulación

**EFECTIVIDAD DE LA INTERVENCIÓN COMBINADA ENTRE ESTIMULACIÓN TRANSCRANEAL POR CORRIENTE DIRECTA Y ESTIMULACIÓN COGNITIVA EN DEMENCIA: UN ESTUDIO DE CASO**

José Ramón Portilla Tellado^1^*, Marta Usúa Calabuig^1^, Carlos Núñez Núñez^1^

^1^Centro de Rehabilitación Cerebral CRECER, 41002 Sevilla, España

*Correspondencia: joseportillatellado@gmail.com

**Resumen**

**Introducción**: Las enfermedades neurodegenerativas afectan a un alto porcentaje de la población a nivel mundial y los tratamientos actuales tienen una eficacia limitada. La Estimulación Transcraneal por Corriente Directa (tDCS) es un tipo de estimulación cerebral no invasiva, la cual ha mostrado tener efectos positivos para este tipo de enfermedades, en especial combinada con sesiones de estimulación cognitiva. Este estudio presenta el análisis de un caso de Enfermedad de Alzheimer en el que se aplicó un protocolo de estimulación con tDCS combinado con sesiones de estimulación cognitiva. Dicho análisis tiene como objetivo evaluar la mejoría o, en su caso, el mantenimiento de las funciones cognitivas afectadas tras la intervención conjunta de ambas técnicas. **Método**: El paciente es un hombre de 75 años, diagnosticado de Demencia tipo Alzheimer de inicio atípico. Se programó una intervención de 10 sesiones con tDCS, donde las primeras 5 sesiones fueron Sham y las 5 posteriores de estimulación. Se utilizó un protocolo en el que el Ánodo se situó en el punto Fp1 del sistema 10–20 y el Cátodo en el punto Fp2. Se estimuló con una intensidad de 2000 mA. durante 20 minutos, con una rampa de 10 segundos hasta llegar a dicha intensidad. Durante las sesiones, se trabajaron diferentes funciones cerebrales afectadas mediante estimulación cognitiva personalizada. Para la valoración se utilizaron el Test MoCA, TMTy 5-Dígitos antes de comenzar el tratamiento, tras las sesiones Sham y al finalizar la estimulación con tDCS. **Resultados**: Se encontró una mejoría de las funciones alteradas al combinar ambos tipos de estimulación. Esta mejoría se observó de forma cualitativa, no pudiéndose evidenciar con pruebas estadísticas. **Discusión**: La estimulación con tDCS parece beneficiar a la estimulación cognitiva desde un punto de vista clínico. La falta de evidencia estadística puede relacionarse con las pocas sesiones de estimulación aplicadas.

**Palabras Clave**

Estimulación Transcraneal por Corriente Directa (tDCS); estimulación cognitiva; deterioro cognitivo; demencia; estimulación cerebral

**IMPACTO DE LA FALTA DE ESTIMULACIÓN COGNITIVA EN EL DETERIORO COGNITIVO DE UN PACIENTE CON DEPRESIÓN CRÓNICA: UN ESTUDIO DE CASO LONGITUDINAL**

Miguel Cruz Cortés^1,2^*, María del Pilar Garrido^1,2^, María Isabel de la Orden Cazorla^1^

^1^Universidad Internacional de La Rioja (UNIR), 26006 Logroño, España

^2^ISANEP Neuropsicología, Psicoterapia y Logopedia, 41500 Sevilla, España

*Correspondencia: miguelcruz@cop.es

**Resumen**

**Introducción**: La ausencia de actividades cognitivamente estimulantes puede acelerar el deterioro cognitivo en individuos con condiciones psiquiátricas. Este estudio de caso examina la evolución de un paciente con depresión crónica, enfocándose en cómo la falta de estimulación cognitiva podría haber contribuido significativamente a su deterioro neuropsicológico y estructural a lo largo de dos años. **Caso Clínico, Evaluaciones y Resultados**: JM, un hombre de 54 años (2022) con antecedentes de depresión crónica que presenta quejas persistentes en problemas atencionales y de memoria. Las pruebas neuropsicológicas realizadas incluyen la WAIS-IV, WMS, RCFT, TMT, Test de Fluencia, Test D2-R, Test de la torre de Londres y Test de Vocabulario de Boston. Se realiza evaluación neuropsicológica (2022) en la que se muestran déficits en denominación y fluidez verbal y capacidad mnésica, sin evidencia de daño estructural en las imágenes de resonancia magnética (RM). En 2024, se realiza una nueva evaluación neuropsicológica observando un deterioro cognitivo adicional, Muestra alteración en denominación, Fluidez semántica y fonológica, Memoria tanto auditiva como visual, en todas sus modalidades y Planificación con evidencias de daños estructurales significativos en RM en región temporal bilateral a nivel mesial, entorrinal, y neocortical simétrica. A lo largo de este periodo, el paciente no participó en actividades cognitivamente estimulantes lo que se asoció con su deterioro progresivo. **Discusión**: La progresión de los déficits cognitivos en este paciente subraya la importancia de la estimulación cognitiva continua. La ausencia de tales actividades puede acelerar el deterioro estructural y funcional del cerebro, especialmente en individuos con trastornos psiquiátricos crónicos. **Conclusiones**: La evolución de JM demuestra cómo la falta de estimulación cognitiva puede influir en el deterioro cognitivo en pacientes con depresión crónica. Este estudio de caso apoya la inclusión de actividades cognitivas como componente crucial en las estrategias de manejo y seguimiento neuropsicológico.

**Palabras Clave**

deterioro cognitivo; evaluación neuropsicológica; resonancia magnética; depresión crónica; estudio de caso

**EVOLUCIÓN DE LA LECTOESCRITURA EN EL PROCESO DE REHABILITACIÓN DE UN PACIENTE CON DAÑO CEREBRAL SOBREVENIDO**

Natalia Muñoz Vigueras^1,2^*, María del Carmen Martínez Cortés^1,2^, Belén Laureano Rico^1,2^, María Navarro Miras^1,2^, Yael Nelea Velasco Collado^1,2^

^1^Centro de Evaluación y Rehabilitación Neuropsicológica, CerNep, 04120 Almería, España

^2^Universidad de Almería, 04120 Almería, España

*Correspondencia: logoinnat@gmail.com

**Resumen**

**Introducción/Objetivos**: El objetivo de este estudio es analizar la evolución de las habilidades de lectura y escritura de un paciente con daño cerebral adquirido post ictus. **Método**: *Diseño*. Se llevó a cabo un estudio longitudinal de caso único. *Participante*. El participante fue un adulto de 40 años, que sufrió un infarto cerebral isquémico por disección de la arteria carotídea en el año 2015, y que desde entonces ha recibido rehabilitación para abordar los trastornos del lenguaje secundarios al daño cerebral. *Procedimiento*. El paciente fue evaluado de manera regular, con el objetivo de controlar su evolución en diferentes áreas del lenguaje. Específicamente en las funciones de lectura y escritura. Las evaluaciones se realizaron de forma continua desde el año 2015 hasta la actualidad, el año 2024. *Materiales*. Se utilizó la Batería para la evaluación de los trastornos afásicos (BETA). **Resultados**: Los resultados obtenidos indicaron que, tanto en la función de lectura, como en la escritura, se observa un patrón similar de evolución. Post ictus, los resultados obtenidos fueron los más bajos. Sin embargo, con la asistencia regular a terapia, se observó una mejora significativa, alcanzando el paciente su puntuación máxima en 2019. Posteriormente, en las evaluaciones realizadas en 2021, se observó un declive significativo en los resultados, coincidiendo con el período post pandemia. Finalmente, en la evaluación realizada en el año 2024, se ha observado una mejora general significativa en la capacidad de lectoescritura del paciente. **Conclusiones**: El estudio revela que la terapia logopédica y neuropsicológica tiene un impacto positivo en la recuperación de la lectoescritura del paciente a lo largo de los años. Además, pone en valor la importancia de un tratamiento sin interrupción, para el mantenimiento de los objetivos.

**Palabras Clave**

lectoescritura; rehabilitación; evolución; terapia; ictus y lenguaje

**EVOLUCIÓN DE LOS DOMINIOS DEL LENGUAJE EN PACIENTES CON DAÑO CEREBRAL SOBREVENIDO: ANÁLISIS COMPARATIVO DE DOS PACIENTES CON AFASIA**

Natalia Muñoz Vigueras^1,2^*, María del Carmen Martínez Cortés^1,2^, Belén Laureano Rico^1,2^, María Navarro Miras^1,2^, Yael Nelea Velasco Collado^1,2^

^1^Centro de Evaluación y Rehabilitación Neuropsicológica, CerNep, 04120 Almería, España

^2^Universidad de Almería, 04120 Almería, España

*Correspondencia: logoinnat@gmail.com

**Resumen**

**Introducción/Objetivos**: Este estudio evalúa y compara la evolución de dos pacientes con daño cerebral adquirido post ictus con afasia, centrándonos en los dominios de comprensión y producción oral, lectura, escritura, semántica y juicio gramatical. **Método**: *Diseño*. Se presenta un estudio longitudinal *Participante*. Dos pacientes adultos de 40 y 63 años con afasia y hemiparesia como secuela de un ictus en estado crónico: pacientes A y B. *Procedimiento*. Se ha llevado a cabo una evaluación de las habilidades lingüísticas en los años 2021 y 2024. *Materiales*. Se utilizó la Batería para la Evaluación de los Trastornos Afásicos (BETA). **Resultados**: *Paciente A.* 1- Mejora en las tareas de: comprensión oral, producción oral, nombrado de letras, lectura de palabras y pseudopalabras; señalar el diferente; emparejamiento oración hablada-dibujo. 2- Empeora en las tareas de: decisión léxica-visual; señalar la letra y dictado de palabras con ortografía arbitraria; emparejamiento definición-palaba; juicio gramatical. *Paciente B*. 1- Mejora en las tareas de: comprensión oral; denominación de objetos y fluidez verbal, denominación de objetos y fluidez verbal; lectura de palabas y pseudopalabras; escritura; emparejamiento definición-palabra; emparejamiento oración hablada-dibujo. 2- Empeora en las tareas de: en denominación de acciones; nombrado de letras; asociación semántica; en juicio gramatical. **Conclusiones**: Las evaluaciones en pacientes con afasia post-ictus son esenciales para personalizar intervención de neurorrehabilitación del lenguaje. El paciente B mostró mejoras en comparación con el paciente A, resaltando la variabilidad en la recuperación post-ictus. Estos resultados subrayan la necesidad de un enfoque individualizado en la rehabilitación del lenguaje.

**Palabras Clave**

afasia; evolución; rehabilitación; BETA

**EVALUACIÓN ATENCIONAL Y EJECUTIVA MEDIANTE REALIDAD VIRTUAL DE MENORES DE ZONAS DE ALTO RIESGO SOCIOECONÓMICO DE CIUDAD DE GUATEMALA**

Alejandro García Crespín^1^*, Pablo Rodríguez Prieto^1^, Joaquín A. Ibáñez Alfonso^1^

^1^Laboratorio de Neurociencia Humana, Departamento de Psicología, Universidad Loyola Andalucía, 14004 Córdoba, España

*Correspondencia: agarciacrespin@al.uloyola.es

**Resumen**

El presente estudio tiene como objetivo analizar el rendimiento de las funciones ejecutivas y atencionales en menores residentes en las zonas de alto riesgo de Ciudad de Guatemala utilizando herramientas innovadoras mediante realidad virtual. Además, también analiza el impacto del contexto socioeconómico, la exposición a la violencia y las dificultades de salud mental (ansiedad y depresión) en el desempeño de estos procesos cognitivos. Por último, a nivel más específico, identifica si existen diferencias en los resultados de las pruebas entre los menores normativos y no normativos (repetidores o con algún diagnóstico o necesidad educativa específica). Se analizaron los datos de 352 participantes con un rango de edad entre los 10–16 años. Los datos fueron recogidos a través de los instrumentos de realidad virtual Nesplora Aula School (aptitudes relacionadas con la atención) y Nesplora Ice Cream (para medir las funciones ejecutivas). Así mismo, se utilizaron diversos cuestionarios con los que se recogió información sociodemográfica familiar, niveles de exposición a la violencia y sintomatología relacionada con ansiedad y depresión. Los principales resultados indican un rendimiento por debajo de la normatividad en las pruebas destinadas a medir la atención y funciones ejecutivas. Así mismo, en cuanto a las diferencias en el rendimiento entre los menores normativos y no normativos, las principales se encuentran en la dimensión atención sostenida, de la prueba atencional, y en la dimensión planificación de la prueba de las funciones ejecutivas, teniendo un mayor rendimiento los menores normativos. Por último, se muestra que la exposición a la violencia influye de manera negativa en el rendimiento de la atención sostenida. Como conclusión general, vivir en este tipo de contextos influye de forma negativa en el rendimiento de las funciones ejecutivas y atencionales.

**Palabras Clave**

atención; funciones ejecutivas; salud mental; exposición a la violencia; nivel socioeconómico; realidad virtual

**FUNCIONES EJECUTIVAS EN HIJOS E HIJAS DE MUJERES VÍCTIMAS DE VIOLENCIA DE GÉNERO**

Ana Isabel de Luis-Ruiz^1^*, Noelia Pérez-Cámara^1,2^, Esperanza Vergara-Moragues^3^, Andrea Benítez Quintana^1,2^, María Paz García-Navas^1^, Nieves Méndez-Ruiz^1^, Miguel Pérez-García^1,2^, Natalia Hidalgo-Ruzzante^1,4^, Inmaculada Teva^1,4^

^1^Centro de Investigación Mente, Cerebro y Comportamiento (CIMCYC), Universidad de Granada, 18071 Granada, España

^2^Departamento de Personalidad, Evaluación y Tratamiento Psicológico, Facultad de Psicología, Universidad de Granada, 18071 Granada, España

^3^Departamento de Psicología, Universidad de Cádiz, 11001 Cádiz, España

^4^Departamento de Psicología Evolutiva de la Educación y del Desarrollo, Universidad de Granada, 18071 Granada, España

*Correspondencia: anaisabeldelr@gmail.com

**Resumen**

La violencia de género es un problema global de dimensiones epidémicas. En España, la mayor parte de las mujeres víctimas tenían hijos/as presentes durante los episodios de violencia. La exposición a esta violencia es una de las formas más graves de maltrato infantil, y genera numerosas secuelas en la salud y la vida diaria de los menores. El objetivo de este estudio es analizar si existen diferencias en memoria de trabajo, atención sostenida y atención selectiva en niños y niñas de entre 7 y 10 años de edad que han sido expuestos/as a violencia de género en comparación con un grupo no expuesto a este tipo de violencia. A través de un diseño transversal se utilizó un cuestionario sociodemográfico completado por las madres y las subpruebas de memoria de trabajo, coordinación motora (atención selectiva) y ejecución continua (atención sostenida) de la batería BENCI con una muestra de 30 menores de la provincia de Granada. No se encontraron diferencias significativas entre el grupo de menores expuestos a violencia de género y el grupo no expuesto a este tipo de violencia en memoria de trabajo, atención sostenida y atención selectiva. Futuras investigaciones deberían ampliar el tamaño de la muestra, observar la influencia de factores mediadores y examinar las posibles diferencias según las características de la violencia. Esto permitirá diseñar intervenciones terapéuticas y programas de estimulación cognitiva específicos para reparar el impacto de la exposición a la violencia.

**Palabras Clave**

Violencia de género; funciones ejecutivas; infancia; exposición; memoria de trabajo; atención

**DESARROLLO DE LAS FUNCIONES EJECUTIVAS ENTRE INFANCIA MEDIA Y ADOLESCENCIA: ESTUDIO LONGITUDINAL EN POBLACIÓN CUBANA**

Rafael Segundo Marcos^1,2^*, Eliana González Rodríguez^3^, Klency González Hernández^3^, María Teresa Daza González^1,2^

^1^Departamento de Psicología, Universidad de Almería, 04120 Almería, España

^2^Centro de Investigación para el Bienestar y la Inclusión Social (CIBIS), Universidad de Almería, 04120 Almería, España

^3^Departamento de Psicología, Universidad de La Habana, 10400 La Habana, Cuba

*Correspondencia: rsm556@ual.es

**Resumen**

Las Funciones Ejecutivas (FFFE) son un conjunto de procesos cognitivos esenciales en nuestra vida diaria, que nos permiten focalizarnos en tareas concretas para resolver problemas y planificar acciones específicas para la consecución de objetivos. Las FFEE han mostrado evidencias de desarrollo desde la infancia temprana hasta la edad adulta. Sin embargo, pocos estudios han examinado específicamente el período comprendido entre la infancia media y la adolescencia, una etapa crítica en la que se producen importantes cambios neurocognitivos y socio-emocionales, además de la intervención de factores contextuales (por ejemplo, cambio de etapa educativa) que pueden influir en su desarrollo. Este estudio exploró las diferencias relacionadas con la edad en el rendimiento de las FFEE en 28 participantes cubanos (14 niños y 14 niñas), quienes fueron evaluados en dos momentos distintos: *T1*, a los 11 años de edad, cuando cursaban 5o y 6o de Educación Primaria; y *T2*, a los 15 años, cuando cursaban 3o y 4o de Educación Secundaria Obligatoria. Se empleó una batería de pruebas informatizadas para evaluar la memoria de trabajo (*tarea tipo Sternberg y 2-Back*), la flexibilidad cognitiva (*tarea Número-Letra*), el control inhibitorio (*Attention Network Test*) y la planificación (*Torre de Hanoi*). Los resultados revelaron mejoras relacionadas con la edad en el rendimiento de las dos tareas de memoria de trabajo, de flexibilidad cognitiva y planificación. No obstante, no se observaron cambios en el desempeño de la tarea de control inhibitorio. Nuestros hallazgos, respaldados por estudios previos, destacan la importancia de estudiar el desarrollo de las Funciones Ejecutivas en este período crítico para obtener una visión más integral de las diferencias relacionadas con la edad en estas habilidades.

**Palabras Clave**

funciones ejecutivas; desarrollo cognitivo; infancia media; adolescencia; factores contextuales

**USO DE LA REALIDAD VIRTUAL COMO TRATAMIENTO COGNITIVO PARA LA ESCLEROSIS MÚLTIPLE: UNA REVISIÓN SISTEMÁTICA**

Laura López Bueno^1^*, Joaquín A. Ibáñez Alfonso^1^

^1^Departamento de Psicología, Universidad Loyola Andalucía, 14004 Córdoba, España

*Correspondencia: llopezbueno@al.uloyola.es

**Resumen**

La esclerosis múltiple (EM) es una enfermedad crónica neurodegenerativa que afecta al sistema nervioso central, considerándose una de las principales causas de discapacidad neurológica no traumática en adultos jóvenes. Los síntomas se centran en déficits en las funciones motoras y cognitivas. Esta revisión sistemática rápida tiene como objetivo actualizar las evidencias disponibles sobre el impacto de las herramientas de realidad virtual en la mejora de la función cognitiva de los pacientes con EM. Se realizó una búsqueda en las bases de datos PubMed y Web of Science de estudios científicos publicados entre 2019 y 2023, con la ecuación palabras clave “Virtual Reality” AND “Multiple Sclerosis” AND Cognit* AND (Rehabilitation OR Treatment), teniendo en cuenta criterios como que fuesen pacientes adultos con EM, y el tipo de estudio que fuese ensayo clínico y aleatorizado o estudio piloto. Tras la lectura y cribado de los estudios encontrados, se seleccionaron un total de 6 para esta revisión. Los resultados muestran que el uso de la realidad virtual como herramienta para la rehabilitación en pacientes con EM tiene efectos positivos, sobre todo en dominios cognitivos como memoria, atención, funciones ejecutivas y visoespaciales, por lo que la evidencia disponible actual la presenta como una herramienta válida para este tipo de tratamientos encaminados a mejorar la funcionalidad y calidad de vida de las personas con EM.

**Palabras Clave**

esclerosis múltiple; realidad virtual; rehabilitación cognitiva; cognición; tratamiento; nuevas tecnologías

**HABILIDADES VISOCONSTRUCTIVAS Y VISOESPACIALES EN MUJERES SUPERVIVIENTES DE VIOLENCIA DE GÉNERO**

María de la Paz García-Navas Menchero^1,2^*, Andrea Benítez Quintana^1,3^, Noelia Pérez Cámara^1,3^, María Pérez González^1,3^, Inma Garrido León^1^, Ana Isabel de Luis Ruíz^1^, Carmen Fernández Fillol^1^, Miguel Pérez García^1,3^, Natalia Hidalgo Ruzzante^1,2^

^1^Centro de Investigación Mente, Cerebro y Comportamiento (CIMCYC), 18071 Granada, España

^2^Departamento de Psicología Evolutiva y de la Educación de la Universidad de Granada, 18071 Granada, España

^3^Departamento de Personalidad, Evaluación y Tratamiento Psicológico de la Universidad de Granada, 18071 Granada, España

*Correspondencia: mpgm0016@gmail.com

**Resumen**

**Introducción**: A nivel mundial 1 de cada 3 mujeres han sufrido o sufren violencia sexual, física y/o psicológica por parte de su pareja o expareja (OMS, 2021). Derivado de la situación de violencia, las mujeres supervivientes presentan multitud de consecuencias para su salud. En los últimos años, el interés por el estudio de las alteraciones neuropsicológicas en esta población ha aumentado. En relación a estas, si bien se ha encontrado que estas mujeres presentan dificultades en habilidades visoconstructivas, aún los estudios al respecto son escasos. **Objetivo**: El objetivo es estudiar si existen diferencias en las habilidades visoconstructivas y visoespaciales en mujeres que han sufrido violencia de género (VG), en comparación a mujeres que no la han sufrido. **Método**: La muestra consta de dos grupos, uno con 58 mujeres supervivientes (MVG), y otro con 43 mujeres que no han sufrido violencia de género (MnVG). En ambos grupos se administró el test del. **Resultados**: Se encontraron diferencias significativas entre ambos grupos (*p* < 0,001; *t* = –2,806). Las mujeres supervivientes (MVG) obtuvieron una menor puntuación en la prueba del Reloj (*M* = 8,67, *DT* = 2,03) en comparación a las mujeres que no han sufrido violencia (MnVG) (*M* = 9,56, *DT* = 1,09). **Conclusiones**: Los resultados evidencian que haber sufrido VG se asocia con un peor rendimiento en las pruebas que miden habilidades visoconstructivas y visoespaciales, en comparación con mujeres que no han sufrido VG. La diferencia de puntuaciones podría sugerir ciertas dificultades leves en la planificación y organización espacial de la información. Sin embargo, es necesario ampliar la investigación sobre el funcionamiento neuropsicológico de esta población para esclarecer dichas diferencias.

**Palabras Clave**

violencia de género; funciones neuropsicológicas; habilidades visoconstructivas; habilidades visoespaciales

**LA RELACIÓN ENTRE LA COORDINACIÓN TEMPORAL Y EL DESARROLLO SOCIOCOGNITIVO EN NIÑOS SORDOS**

Laura Fernández García^1,2^*, Nicholas A. Smith^3^, Jessica Phillips-Silver^4^, María Teresa Daza González^1,2^

^1^Centro de Investigación para el Bienestar y la Inclusión Social, 04120 Almería, España

^2^Departamento de Psicología, Almería, España

^3^Department of Speech, Language and Hearing Sciences, University of Missouri, Columbia, MO 65211, USA

^4^Growing Brains, Washington, D.C., USA

*Correspondencia: lfg403@ual.es

**Resumen**

**Introducción y Objetivos**: En la mayoría de los casos de sordera, la pérdida auditiva afecta a la calidad de las interacciones sociales tempranas. En consecuencia, en las interacciones sociales se suelen emplear expresiones más cortas y, los padres utilizan menos referencias sobre los estados mentales. Dentro de las interacciones sociales, un aspecto menos estudiado es la capacidad de coordinación temporal donde adultos y niños mantienen una conversación contingente y con mínimas interrupciones. Por lo que en el presente estudio se exploró cómo tienen lugar la coordinación temporal durante las interacciones adultos-niños con sordera. Y, si esta habilidad para coordinarse temporalmente podría estar relacionada con la teoría de la mente (ToM) de estos niños. Método: Participaron un total de 15 niños con sodera de moderada a profunda (Edad media = 8,40, DT = 1,35, 40% niñas), cuyo principal sistema de comunicación fue el lenguaje oral. Como medida de la coordinación temporal, se registraron las conversaciones entre los niño-adulto familiar (su logopeda), y niño-adulto desconocido (la examinadora). Mientras que para evaluar la ToM se utilizó una tarea informatizada basada en el paradigma de cambio de localización que implica comprensión de falsas creencias. **Resultados y Conclusiones**: Los resultados parecen indicar que, a pesar de la condición de sordera, todos los niños fueron capaces de coordinarse temporalmente con los adultos familiares, pero no con el adulto desconocido. Además, los tiempos de respuesta de los niños cuando interactuaron con los adultos desconocidos correlacionaron positivamente con las puntuaciones de ToM. De manera que, cuando interactúan con adultos con los que no están acostumbrados a relacionarse, aquellos niños que tienen una mejor habilidad de ToM, parecen tomarse más tiempo para responder. Podemos concluir por tanto que, la habilidad de coordinación temporal durante las interacciones adulto-niño sordo parece depender de diversos aspectos, como la familiaridad del adulto.

**Palabras Clave**

coordinación temporal; teoría de la mente; sincronización; sordera; infancia media

**EFECTOS DE UN PROGRAMA DE ENTRENAMIENTO DE FUERZA Y AERÓBICO SOBRE LA FUNCIÓN COGNITIVA Y FÍSICA DE UN PACIENTE CON TRAUMATISMO CRANEOENCEFÁLICO**

Alejandro Olivares Balbuena^1^, María Navarro Miras^1^, María del Carmen Martínez Cortes^1^*, Belén Laureano Rico^1^, Natalia Muñoz Vigueras^1^, Yael Velasco Collado^1^

^1^Centro de Evaluación y Rehabilitación Neuropsicológica, CerNep-CiBis, Universidad de Almería, 04120 Almería, España

*Correspondencia: maicamc1@gmail.com

**Resumen**

**Introducción/Objetivos**: Se presentan los resultados en rendimiento cognitivo y físico de un programa de entrenamiento de fuerza y aeróbico en un paciente con daño cerebral adquirido-crónico por traumatismo craneoencefálico. **Método**: Diseño. Se llevó a cabo una evaluación pre-post intervención. Participante. paciente adulto de 40 años con afectación ejecutiva. Procedimiento. La intervención se implementó a lo largo de 5 semanas con 5 sesiones semanales de 45 minutos. En total, se completaron 17 sesiones, de las cuales 4 fueron grupales y 13 individuales. Las sesiones de intervención se organizaron en torno al entrenamiento de fuerza, y a ejercicios de movilidad y de estabilización/equilibrio. **Materiales**: Para la evaluación de la esfera cognitiva se emplearon los tests de MOCA, D2, Fluencia verbal y Dígitos Directos e Inversos. Para la función física se emplearon diferentes medidas: Prueba de prensión manual con handgrip, Sit to Stand, Get Up and Go, Test de velocidad 4 metros y Test de velocidad de 10 metros, Prueba de 6 minutos marcha, el Biceps Curl Test y test Sit and Reach. Resultados: MOCA(Pre:26/Post:29), Fluencia verbal semántica (Pre:22/Post:27) Fluencia verbal fonética (Pre:24/Post:22), Dígitos directos (Pre:5/Post:5), Dígitos Inversos (Pre:4/Post:5), D2: mejoría en concentración, velocidad de trabajo así como en precisión. El paciente evidenció mejoras significativas en 3 de las 8 pruebas físicas evaluadas. Mostró una mayor distancia recorrida en el test de 6 minutos marcha (601,3 m <639,1 m), así como más centímetros en la prueba de flexibilidad Sit and reach (–20,75 cm <–15,25 cm), asimismo se mostró un mayor número de repeticiones en el Biceps Curl Test (19 <22). De esta manera se vieron mejoras en la capacidad cardiorrespiratoria, la flexibilidad de los miembros inferiores y la fuerza de los miembros superiores. **Conclusiones**: El entrenamiento de fuerza y aeróbico intensivo resultó ser eficaz para el paciente ya que los resultados de la intervención mostraron mejorías en diferentes dominios cognitivos y físicos.

**Palabras Clave**

daño cerebral adquirido; ejercicio de fuerza; aeróbico; ictus; atención; fluencia verbal

**ANSIEDAD PERINATALY SINTOMATOLOGÍA PSICOPATOLÓGICA: FACTORES DE RIESGO PSICONEUROINMUNOLÓGICOS RELACIONADOS**.

Gloria Sánchez Torices^1^*, Jesús Hijona Elósegui^2^, María Isabel Peralta Ramírez^3^, Teresa Sánchez Gutiérrez^4^

^1^Facultad de Ciencias de la Salud, Universidad Internacional de la Rioja, 26006 Logroño, España

^2^Unidad Clínica de Ginecología y Obstetricia del Hospital Universitario de Jaén, 23071 Jaén, España

^3^Departamento de Personalidad, Evaluación y Tratamiento Psicológico, Universidad de Granada, 18071 Granada, España

^4^Departamento de Psicología, Universidad de Córdoba, 14071 Córdoba, España

*Correspondencia: gloriast@correo.ugr.es

**Resumen**

La ansiedad perinatal se considera un predictor de complicaciones durante el embarazo y el parto. Ciertos factores sociodemográficos, síntomas psicopatológicos y cambios biofisiológicos sufridos durante el embarazo, predisponen para la ansiedad perinatal. El objetivo del estudio fue analizar la relación entre ansiedad perinatal, factores sociodemográficos, factores psiconeuroinmunológicos [(cortisol e interleucina-6 (IL-6)] y síntomas psicopatológicos en embarazadas. Se seleccionaron n = 249 mujeres embarazadas (33,4 ± 4,8) y se utilizaron los instrumentos: (1) Cuestionario sociodemográfico, (2) Perinatal Anxiety Screening Scale (PASS), (3) Symptom Assessment-45 Questionnaire (SA-45). Se analizaron muestras de sangre para obtener el cortisol e IL-6 y análisis capilar mediante método ELISA para obtener valores retroactivos de cortisol. Se compararon puntuaciones del PASS y factores psiconeuroinmunológicos con el estadístico t de student y r de Pearson, y psicopatología y grupos de ansiedad con U de Mann-Whitney. El nivel de significación fue *p* ≤ 0,05 y se usó SPSS versión 25,0. Los resultados mostraron que el 49,8% de la muestra presentaba ansiedad clínica, con puntuaciones significativamente más altas en síntomas psicopatológicos [Hostilidad (U = 4097,5, *p* < 0,001), Somatización (U = 3895,0, *p* < 0,001), Depresión (U = 2768,5, *p* < 0,001), Compulsión (U = 3075,0, *p* < 0,001), Ansiedad (U = 2637,5, *p* < 0,001), Sensibilidad (U = 2768,5, *p* < 0,001), Ansiedad fóbica (U = 4867,0, *p* < 0,001), Ideación paranoide (U = 3270,5, *p* < 0,001), Psicoticismo (U = 4567,0, *p* < 0,001)] y total (U = 1,526, *p* < 0,001). Se observaron mayores niveles de cortisol en sangre (t = –1.7, *p* = 0,048) sin diferencias en IL-6 y cortisol capilar, una relación inversa entre cortisol en sangre e IL-6 (r = –0,2, *p* = 0,003), una relación directa entre cortisol en sangre y ansiedad fóbica (r = 0,1, *p* = 0,04), una relación inversa entre cortisol capilar y somatización (r = –0,2, *p* = 0,009) y una relación directa entre IL-6 y somatización (r = 0,1, *p* = 0,50). Se concluye que las mujeres embarazadas con ansiedad clínica autoinformada y altos niveles de factores psiconeuroinmunológicos presentan también sintomatología psicopatológica.

**Palabras Clave**

ansiedad; embarazo; psicopatología; cortisol; citoquinas

**MODIFICACIÓN DEL SESGO DE APROXIMACIÓN-EVITACIÓN PARA EL TRATAMIENTO DEL EXCESO DE PESO**

Raquel Vilar López^1^*, Lucía Solier López^1^, Raquel González González^1^, Francisco Javier Pérez Comino^1^, Andrea Bernat Villena^1^, Luz Stella Algarra López^1^, Alfonso Caracuel^1^, Marta Becerra Losada^1^

^1^Centro de Investigación Mente, Cerebro y Comportamiento 1, 18071 Granada, España

*Correspondencia: rvilar@ugr.es

**Resumen**

La prevalencia de la obesidad y el sobrepeso continúa en aumento, convirtiéndose en una crisis de salud pública a nivel mundial. Los tratamientos actuales, como los cambios en el estilo de vida, la cirugía bariátrica y los tratamientos farmacológicos, han demostrado ser poco eficaces a largo plazo. Una alternativa prometedora es el entrenamiento cognitivo para modificar el sesgo de aproximación-evitación, que afecta las respuestas automáticas hacia la comida. El objetivo de este estudio es evaluar la efectividad del entrenamiento de dicho sesgo durante una semana con la aplicación TiltTask para reducir el sesgo de aproximación hacia alimentos no saludables en personas con sobrepeso u obesidad. Se llevó a cabo un ensayo controlado aleatorizado de doble ciego con tres grupos: experimental, placebo y control. Los tres grupos recibían el tratamiento habitual para el exceso de peso, consistente en una dieta individualizada y pautas de ejercicio físico. Los resultados del ANOVA mixto 3x2 mostraron diferencias significativas en la reducción del sesgo de aproximación en el grupo experimental en comparación con los grupos placebo y control (F = 3,992, *p* = 0,021). Además, los tamaños del efecto mostraron un efecto clínicamente significativo solo en el grupo experimental (*d* = –0,36) frente a los otros dos grupos (*d* = –0,02 y 0,24). Estos hallazgos sugieren que las intervenciones cognitivas pueden ser un complemento valioso a los tratamientos tradicionales para la obesidad.

**Palabras Clave**

exceso de peso; obesidad; entrenamiento cognitivo; sesgo de aproximación-evitación; tilt task

**EFECTIVIDAD DE UNA INTERVENCIÓN COGNITIVA INTEGRAL PARA EL TRATAMIENTO DEL EXCESO DE PESO**

Andrea Bernat Villena^1^*, Francisco Javier Pérez-Comino^1^, Marta Becerra-Losada^1^, Luz Stella Algarra López^1^, Alfonso Caracuel^1^, Raquel González González^1^, Lucía Solier López^1^, Raquel Vilar López^1^

^1^Centro de Investigación, Mente, Cerebro y Comportamiento, Universidad de Granada, 18071 Granada, España

*Correspondencia: andreabernat@ugr.es

**Resumen**

El exceso de peso (EP) ha aumentado en todo el mundo, alcanzando ya al 60% de los adultos. Existen diferentes tratamientos, como el asesoramiento nutricional, el ejercicio físico, la cirugía bariátrica, los fármacos y la terapia cognitivo-conductual, pero su eficacia a largo plazo es limitada. La neurociencia ha revelado alteraciones cognitivas y cerebrales en las personas con EP, lo que ha motivado la investigación en estrategias de entrenamiento cognitivo que mejoren la adherencia a hábitos alimentarios y de ejercicio saludables. El objetivo del estudio fue evaluar la eficacia de un programa cognitivo integral para el tratamiento del EP que combina la Modificación del Sesgo de Aproximación-Evitación, el Entrenamiento en Control Inhibitorio, la Implementación de Intenciones y el Pensamiento Episódico Futuro. En esta comunicación se presentan los resultados de los 45 participantes con EP que, de modo aleatorio, no fueron asignados al grupo experimental sino a los grupos placebo y control, ambos sin tratamiento cognitivo activo. Sin embargo, una vez finalizado el estudio, recibieron el programa cognitivo integral (tratamiento activo). Los resultados demuestran la efectividad del programa cognitivo, con reducciones estadísticamente significativas pre-post tratamiento del Índice de Masa Corporal (IMC) (*p* = 0,025), de la valoración de alimentos poco saludables (*liking*) (*p* = 0,001) y del tiempo diario sentado (*p* = 0,003), así como un incremento de la adherencia a la dieta mediterránea (*p* = 0,005) y de ejercicio físico moderado (*p* = 0,027). Además, aplicar las 4 intervenciones de forma conjunta produjo mayor efecto del tratamiento en comparación con la evidencia previa sobre la implementación de cada una de forma separada en IMC (*d* = 0,345), actividad física (*d* = 0,350), dieta mediterránea (*d* = 0,446), tiempo sentado (*d* = 0,5) y *liking* (*d* = 0,886). Estas mejoras no se observaron cuando los participantes recibieron el entrenamiento inactivo. En conclusión, el entrenamiento cognitivo dirigido a las alteraciones descritas en personas con EP resulta eficaz para mejorar el éxito del tratamiento.

**Palabras Clave**

exceso de peso; entrenamiento cognitivo; modificación del sesgo de aproximación-evitación; control inhibitorio; implementación de intenciones y pensamiento episódico futuro

**ENTRENAMIENTO COGNITIVO MULTICOMPONENTE PARA TRATAMIENTOS DE PÉRDIDA DE PESO**

Francisco Javier Pérez Comino^1^, Alfonso Caracuel^1^, Raquel González González^1^, Marta Becerra Losada^1^, Andrea Bernat Villena^1^, Raquel Vilar López^1^, Luz Stella Algarra López^1^, Lucía Solier López^1^*

^1^Centro de Investigación, Mente, Cerebro y Comportamiento (CIMCYC), 18071 Granada, España

*Correspondencia: luciasl@ugr.es

**Resumen**

El exceso de peso (EP; sobrepeso y obesidad) ha alcanzado niveles pandémicos. Los tratamientos habituales centrados en dieta y actividad física tienen un bajo grado de éxito a largo plazo. Nuevos enfoques basados en la neuropsicología son prometedores para mejorar aspectos ejecutivos deficitarios en las personas con EP. El objetivo del estudio fue mejorar los procesos cognitivos para aumentar la adherencia al tratamiento habitual con el objetivo de reducir el Índice de Masa Corporal (IMC) y mantener la pérdida de peso en el tiempo. La muestra fue de 148 personas con EP (85,1% mujeres), con edad media de 44 años (DT = 6,78), educación media de 15 años (DT = 5,34) e IMC medio de 31,61 (DT = 4,01). Los participantes fueron asignados aleatoriamente al grupo experimental (intervención cognitiva); al grupo sham (intervención cognitiva placebo); o al grupo de control. Los 3 grupos recibieron pautas individualizadas de dieta y ejercicio físico. El grupo experimental recibió 4 intervenciones de entrenamiento cognitivo (sesgo de aproximación- evitación, control inhibitorio, implementación de intenciones, pensamiento episódico futuro) secuenciales a lo largo de 4 semanas. El grupo sham recibió versiones placebo de dichas intervenciones. Los resultados de un ANOVA mixto de medidas repetidas mostraron cambios estadísticamente diferentes en el IMC de los grupos (F = 4,909, *p* = 0,009). Las pruebas t de Student indicaron un mayor % de pérdida de peso en el grupo experimental respecto al placebo a lo largo del tiempo: basal- postratamiento (t = 2,291, *p* = 0,012), basal-seguimiento 3 meses (t = 1,981, *p* = 0,025), basal-seguimiento 6 meses (t = 1,973, *p* = 0,026). Además, en el grupo experimental un mayor % de personas alcanzaron una pérdida de peso clínicamente significativa mantenida a los 6 meses (χ^2^ = 9,700, *p* = 0,008). En conclusión, incluir un programa de entrenamiento cognitivo multicomponente mejora la eficacia del tratamiento habitual para perder peso y mantener los resultados. Estos resultados avalan la necesidad de abordar los aspectos cognitivos en los tratamientos para personas con EP.

**Palabras Clave**

sesgo de aproximación-evitación; control inhibitorio; implementación de intenciones; pensamiento episódico futuro; pérdida de peso; exceso de peso

**TEMPERAMENTO DEL BEBÉ Y NEURODESARROLLO A LOS DOS AÑOS**

Javier De Echarri Lorente^1^*, Miguel Ángel Baos González^1,2^, Carolina Mariño Narváez^2^, Borja Romero González^3^, María Isabel Peralta Ramírez^1,2^

^1^Facultad de Psicología, Universidad de Granada, 18011 Granada, España

^2^Centro de Investigación Mente, Cerebro Y Comportamiento (CIMCYC), 18071 Granada, España

^3^Facultad de Educación de Soria, Universidad de Valladolid, 42004 Soria, España

*Correspondencia: javierdeecharri@gmail.com

**Resumen**

**Introducción**: Entendiendo por temperamento las diferencias individuales en el comportamiento, así como reacciones emocionales que se mantienen relativamente estables y que aparecen en los primeros años de vida. El temperamento es clave en el desarrollo del niño ya que una respuesta diferente ante los retos comunes del desarrollo deriva en interacciones diferentes con el ambiente, llevando a la heterogeneidad en el desarrollo. Sin embargo, a nivel de neurodesarrollo ha sido poco estudiado cómo puede interaccionar con los primeros momentos de vida, sino más bien en su relación con problemáticas como el autismo o el TDAH. Por ello, el objetivo del presente estudio es analizar la interacción que tiene el temperamento del bebé en su nivel de neurodesarrollo a los dos años.** Método**: Se evaluaron a 59 niños y niñas sanos a los dos años de la cohorte Childstress a través de la escala Bayley-III para obtener una medida del neurodesarrollo y simultáneamente las madres informaron de su temperamento a través de la escala EAS. **Resultados**: Se encuentra una relación inversa entre la escala de sociabilidad y las dimensiones de desarrollo de comunicación receptiva (*r*: –0,336; *p*: 0,009) y motricidad fina (*r*: –0,367; *p*: 0,004). Lo que significa que a mayor sociabilidad, habrá un menor desarrollo tanto de comunicación receptiva como de motricidad fina. Además, existe una relación negativa entre la escala de timidez y la motricidad gruesa (*r*: –0,324; *p*: 0,012). **Conclusiones**: A mayor timidez, se encuentran niveles más bajos de motricidad gruesa. No se encontró relación con las demás dimensiones de temperamento y neurodesarrollo. Los resultados muestran que hay relación entre el temperamento del niño (informado por progenitores) y el neurodesarrollo, siendo una de las variables que guía las oportunidades que tiene cada niño en los ambientes en los que se desarrolla.

**Palabras Clave**

temperamento; neurodesarrollo; infancia; Bayley-III

**¿HAY DIFERENCIAS ENTRE MUJERES Y HOMBRES QUE CONSUMEN CANNABIS EN LA REGULACIÓN EMOCIONAL E IMPULSIVIDAD?**

Agar Marín Morales^1,2^*, Bella M. González Ponce^1,3,4^, Miguel Pérez García^2,5^, José Carmona Márquez^1,3^

^1^Departamento de Psicología Clínica y Experimental, Universidad de Huelva, 21071 Huelva, España

^2^Centro de Investigación Mente, Cerebro y Comportamiento (CIMCYC), Universidad de Granada, 18071 Granada, España

^3^Centro de Investigación en Recursos Naturales, Salud y Medio Ambiente, Universidad de Huelva, 21071 Huelva, España

^4^Departamento de Psicología y Antropología, Universidad de Extremadura, 06006 Badajoz, España

^5^Departamento de Personalidad, Evaluación y Tratamiento Psicológico, Universidad de Granada, 18071 Granada, España

*Correspondencia: agar.marin@dpces.uhu.es

**Resumen**

**Introducción**: Las estrategias de regulación emocional y control de impulsos son cruciales para el funcionamiento psicológico, la salud mental y adaptación social (Gross y Thompson, 2007). Altos niveles de impulsividad y menor uso de regulación emocional se asocian con el consumo de cannabis . Es relevante estudiar estas relaciones según el género para mejorar las intervenciones. **Objetivo**: Analizar la relación entre consumo de cannabis, impulsividad y regulación emocional según el género. **Método**: Muestreo dirigido a poblaciones diana para acceder a muestra comunitaria. Se administraron cuestionarios sobre regulación emocional (ERQ, Gross & John, 2003), impulsividad (UPPS, Whiteside & Lynam, 2001) y consumo de cannabis a 612 jóvenes (18–25 años; 371 hombres, Medad = 20,96, DT = 2,13; 235 mujeres, Medad = 21,15, DT = 2,19). Se realizaron pruebas t de Student para comparar grupos y correlaciones de Pearson para examinar relaciones entre variables. **Resultados**: Se encontraron diferencias significativas en consumo de cannabis t(602) = 2,52, *p* = 0,007, siendo mayor en hombres. También hubo diferencias en la subescala de supresión expresiva del ERQ t(604) = 2,57, *p* = 0,013, y en la subescala de urgencia negativa de la UPPS t(604) = –3,31, *p* = 0,001, con puntuaciones mayores en hombres. El consumo de cannabis se correlacionó positivamente en ambos grupos con supresión expresiva y urgencia negativa (all = *p* < 0,01). **Conclusiones**: Existen diferencias en consumo de cannabis, impulsividad y regulación emocional según el género. Los hombres consumen más cannabis y el consumo se relaciona con mayores niveles de impulsividad y el uso de la estrategia desadaptativa de supresión emocional. Es fundamental personalizar las estrategias de intervención según el género y mejorar las habilidades de regulación emocional y control de impulsos en jóvenes.

